# Notes on the distribution and habitat use of marmosets (Callitrichidae: *Mico*) from south-central Amazonia

**DOI:** 10.5194/pb-10-7-2023

**Published:** 2023-07-28

**Authors:** Rodrigo Costa-Araújo, Giovanna Bergamasco, Christian Roos, Izeni Pires Farias, Tomas Hrbek

**Affiliations:** 1 Primates Genetics Laboratory, German Primate Center, Leibniz Institute for Primate Research, 37077 Göttingen, Germany; 2 Graduate Program in Ecology, Evolution and Biodiversity, São Paulo State University, Rio Claro, 13506-900, Brazil; 3 Gene Bank of Primates, German Primate Center, Leibniz Institute for Primate Research, 37077 Göttingen, Germany; 4 Evolution and Animal Genetics Laboratory, Federal University of Amazonas, Manaus, 69077-000, Brazil; 5 Department of Biology, Trinity University, San Antonio, Texas 78212-7200, United States of America

## Abstract

Currently, 15 species of Amazon marmosets (genus *Mico*) are known to science. The Amazon marmosets occur primarily in southern Brazilian Amazonia, the arc of deforestation, and are among the least studied primates of the neotropics. This is particularly the case for *M. acariensis* and *M. chrysoleucos*, both endemic to the Aripuanã–Sucundurí interfluve, south-central Amazonia. *Mico acariensis* was not studied beyond the species description, and the only information currently available is the pelage colouration of the holotype, inferred coordinates of the type locality, and a field report with two additional localities of occurrence. Regarding *M. chrysoleucos*, in addition to the species description, there are taxonomic reviews, the report of a second occurrence record, and a study on the species range. We provide here new occurrence records that extend the distribution of *M. chrysoleucos*; provide new records for and update the distribution of *M. acariensis*; and propose the existence of a hybrid zone in the Aripuanã–Sucundurí interfluve, i.e. around the known distribution boundaries of *M. acariensis*, *M. chrysoleucos*, and *M. melanurus*, and we also discuss habitat use patterns of Amazon marmosets.

## 1 Introduction

The Amazon marmosets (genus *Mico*) are among the least studied primates of the neotropics. To date, most *Mico* species were subjected solely to taxonomic and some phylogenetic studies essentially based on the morphology of museum specimens from few localities or on genetic data of a few captive individuals (Costa-Araújo et al., 2023a). Therefore, a number of uncertainties still remain concerning the taxonomy, geographic distribution, and phylogenetic relationships of *Mico* species, and the ecology and behaviour of Amazon marmosets remain largely unstudied (Costa-Araújo, 2020).

**Figure 1:**
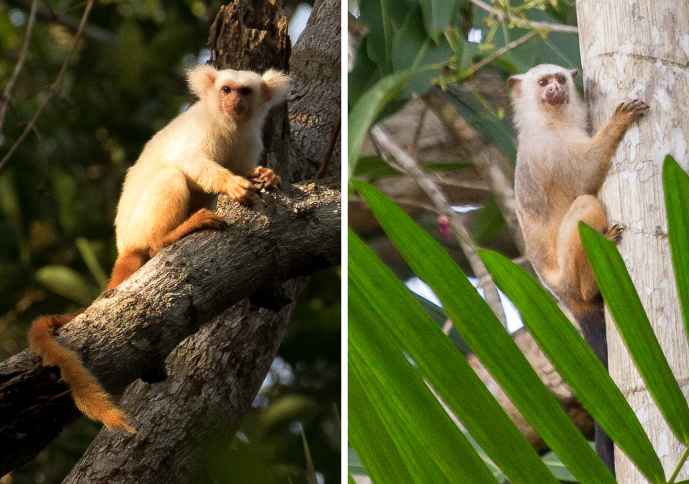
*Mico* *chrysoleucos* (left; photo: Jon Hall) and *Mico acariensis* (right; photo: Diogo Lagrotería).

The Aripuanã–Sucundurí interfluve in south-central Amazonia harbours two endemic *Mico* species: *M. chrysoleucos* (Natterer in Wagner, 1842) and *M. acariensis* (Roosmalen et al., 2000; Fig. 1). *Mico chrysoleucos* was described in 1842, based on pelage patterns of specimens collected by Johann Natterer in Borba (Wagner, 1842, 1847) – which is a town located on the right bank of the Aripuanã River, Borba municipality, Amazonas state, Brazil. *Mico acariensis* was described in 2000 based on pelage colour of an individual obtained by Marc van Roosmalen from a *ribeirinho* on the left bank of Sucundurí River – which was defined as the species type locality (Roosmalen et al., 2000). The Acarí River divides the range of both species, and *M. melanurus* can also be found in a small portion of the extreme south of the Aripuanã–Sucundurí interfluve (Noronha et al., 2008; Rylands and Mittermeier, 2013).

For *M. chrysoleucos*, in addition to the species description, there are taxonomic reviews (see Hershkovitz, 1977) and a note on its occurrence (Silva-Júnior and Noronha, 2000). Further, Silva et al. (2018) restricted the range of *M. chrysoleucos* to the right bank of the Aripuanã and Madeira (and Amazon) rivers, part of the fluvial islands of the Paraná Urariá, and the left bank of the Acarí River. Beyond the species description, *M. acariensis* has not been studied so far. The only information available for this species is the pelage colouration of the holotype, a single occurrence record inferred from the species description, and two additional localities recorded by Noronha et al. (2007).

Here we provide new occurrence records that clarify and extend the distribution of *Mico chrysoleucos*; support the sole occurrence of *M. acariensis* in the Acarí–Sucundurí interfluve; suggest the existence of a contact zone with potential hybridization between *M. acariensis*, *M. chrysoleucos*, and *M. melanurus*; and bring new insights concerning habitat use patterns of Amazon marmosets. Our results derive from an ongoing field-based research programme specifically focused on Amazon and dwarf marmosets (genus *Callibella*).

## 2 Material and methods

During 2015–2018, we carried out 10 field expeditions across the arc of deforestation, southern Amazonia, Brazil, focusing on marmoset detection using playback surveys (see Costa-Araújo et al., 2023b, for details). Species identifications follow the latest taxonomic reviews of neotropical primates (Rylands et al., 2008; Rylands and Mittermeier, 2013) and are based on phenotype or on the locality. The coordinates, originally taken in degrees–minutes–seconds (DMS), were transformed into decimal degrees using the speciesLink converter for geographic coordinates (http://splink.cria.org.br/conversor?criaLANG=en, last access: 25 April 2023) and plotted on a map using QGIS (2023). Formerly available occurrence records of marmosets were obtained from the literature.

No ethics consent was necessary for this research, which nonetheless abided by the *Code of Best Practices for Field Primatology* of the International Primatological Society (http://www.internationalprimatologicalsociety.org/policy-statements-and-guidelines, last access: 25 April 2023) and the “2016 Guidelines of the American Society of Mammalogists for the use of wild mammals in research and education” (Sikes et al., 2016).

## 3 Results and discussion

**Figure 2:**
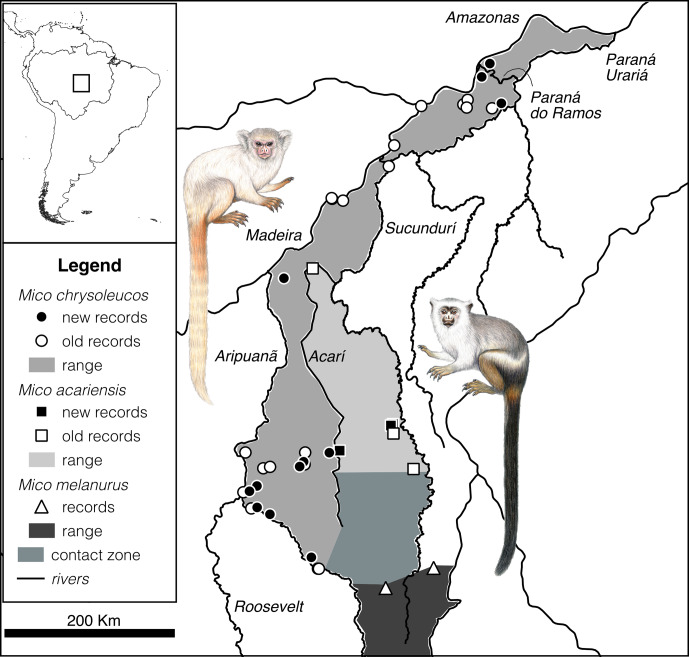
Updated distributions of *Mico chrysoleucos* and *M. acariensis*, based on new occurrence records from field expeditions. The maps show South America, the boundaries of the Amazonia biome, the location of the study area in south-central Amazonia (top left), the distributions of *M. chrysoleucos* and *M. acariensis*, the northern limit of the distribution of *M. melanurus* (see the Supplement​​​​​​​ for coordinates), and the contact zone with potential hybridization between these three species (main map). Stephen Nash's illustrations of *Mico chrysoleucos* (left) and *Mico acariensis* (right).

We provide here 12 new records for *M. chrysoleucos* and 3 new records for *M. acariensis* from the municipalities of Apuí, Maués, Novo Aripuanã, and Urucurituba, southern Amazonia, Amazonas state, Brazil (the Supplement). These records allow us to refine the geographical distribution of *M. chrysoleucos*, confirm the distribution of *M. acariensis*, suggest the existence of a contact zone with potential hybridization between these two species and *M. melanurus* (Fig. 2), and discuss Amazon marmosets' habitat use patterns.

Our new record for *M. chrysoleucos* on the eastern margin of Paraná do Ramos extends the species distribution onto Ilha Tupinambarana, widening its eastern limit for 160 km and increasing the extent of occurrence by 11 850 km ${}^{2}$ . According to this new data, the distribution of *M. chrysoleucos* covers an area of 46 800 km ${}^{2}$ , and it is delimited by the Madeira and Amazon rivers to the north, by the Aripuanã River to the west, by the Paraná Urariá and Acarí River to the east, and by the Maracanã River to the south. According to the investigation by Vanzolini (1993) on the travels of Johann Natterer in Brazil during the 19th century, there are four potential specific localities within Borba municipality where the type specimens might have been collected: “Igarapé do Jaguar”, “sítio de Hilário Goes”, “sítio de Joaquim Nunes Collares”, and “sítio de Joaquim Silva”. The most likely locality for the collection of *M. chrysoleucos* type specimens in Borba is “sítio de Joaquim Nunes Collares”, indicated by Vanzolini (1993) as located on the right margin of Madeira River.

*Mico acariensis* is among the less-known primates of the neotropical region. The species description was based on pelage colouration of a sole infant marmoset, which was obtained from a *ribeirinho*'s household and raised to adulthood in captivity before the species description (Roosmalen et al., 2000). This species was not studied since then; thus, there is little information about the taxonomy and distribution. Nonetheless, the two localities reported by Noronha et al. (2007) and the three new localities reported here confirm *M. acariensis* as the only marmoset in the Acarí–Sucundurí interfluve. Based on these data, the distribution of *M. acariensis* is delimited by the right bank of the Acarí River and the left bank of the Sucundurí River, and it extends south to the BR-230 Rodovia Transamazônica (Trans-Amazonian Highway), covering an area of approximately 17 480 km ${}^{2}$ .

Considering the information available for the distribution of *M. chrysoleucos*, *M. acariensis*, and *M. melanurus*, as well as the geomorphology of the Aripuanã–Sucundurí interfluve, we suggest the existence of a zone of contact between these three species around the headwaters of the Acarí River. There are no physical barriers that would impede the dispersal of *M. acariensis* further south, of *M. chrysoleucos* further east, or of *M. melanurus* further north, beyond the limits currently known for the ranges of these species. In fact, the southernmost record of *M. chrysoleucos* is located above the Acarí River headwaters, at the same latitude as the northernmost record of *M. melanurus*.

Given that hybridization is well known for closely related primate species (Cortés-Ortiz et al., 2019), even for species of contrasting phenotypes (Mourthé et al., 2019) and specifically for marmoset species of the genus *Callithrix* (Malukiewicz, 2019), we expect to find different combinations of hybrids between *M. acariensis*, *M. chrysoleucos*, and *M. melanurus* around the headwaters of the Acarí River. This region is difficult to access, but additional surveys should be carried out to collect records, specimens, and samples to clarify the geographic distribution, the existence of the contact zone, and the population dynamics and hybridization of these three species.

Our records of *M. chrysoleucos* also shed new light on habitat use of Amazon marmosets. Groups of *M. chrysoleucos* were documented (voucher specimen codes RCA 14/INPA 7388, RCA 15/INPA 7389, RCA 16/INPA 7390, stored in the mammal collection of the Instituto Nacional de Pesquisas da Amazônia–INPA–Manaus, Brazil) in areas permanently flooded by white waters – *várzea* forest – of the Amazon River at Paraná do Ramos and in forests permanently flooded by black waters – *igapó* forest – on the left bank of the Acarí River. To our knowledge, these are the first records of any *Mico* species in permanently flooded forests. In fact, these two records of *M. chrysoleucos* and the record of *Cebuella pygmaea* in the *várzea* forest of the Urucu River (Amazonas state, Brazil; Peres, 1993) are the only known records for any marmoset from Amazonia (*Mico*, *Callibella*, and *Cebuella* genera) in permanently flooded forests – in addition to scanty evidence of *Callithrix penicillata* and *Callithrix jacchus* using mangrove habitats in eastern Brazil (see Nowak et al., 2019).

Nonetheless, considering the current evidence of marmosets (*Callithrix penicillata*, *Callithrix jacchus*, *Cebuella pygmaea*, *Mico humeralifer*, *M. melanurus*) using to some extent different types of seasonally flooded forests (Nowak et al., 2019) and permanently flooded forests as here reported, it is possible that marmosets are more often living in such habitats than previously thought. The scarcity of information on such habitat use patterns for marmosets might be due to a research bias, given that flooded forests are more difficult to access.

## Supplement

10.5194/pb-10-7-2023-supplement The supplement related to this article is available online at: https://doi.org/10.5194/pb-10-7-2023-supplement.

## Data Availability

The data of all known occurrence records of and can be found in the Supplement.
