# Supplementary material for: Notes on the distribution and habitat use of marmosets (Callitrichidae: Mico) from south-central Amazonia
Source: Primate Biol. 2023 Jul 28;10(2):7–11. doi: 10.5194/pb-10-7-2023 (PMC10407307; doi:10.5194/pb-10-7-2023)
Supplement: The supplement related to this article is available online at: https://doi.org/10.5194/pb-10-7-2023-supplement. [file pb-10-7-supplement.pdf]

Supplement of Primate Biol., 10, 7–11, 2023
https://doi.org/10.5194/pb-10-7-2023-supplement
© Author(s) 2023. CC BY 4.0 License.

Primate Biology
Open Access

Supplement of

Notes on the distribution and habitat use of marmosets (Callitrichidae: *Mico*) from south-central Amazonia

Rodrigo Costa-Araújo et al.

Correspondence to: Rodrigo Costa Araújo (rcostaaraujo@dpz.eu)

The copyright of individual parts of the supplement might differ from the article licence.

Supplementary material. Occurrence records of *Mico chrysoleucos*, *Mico acariensis* and *M. melanurus* on the Aripuanã–Sucundurí interfluve, south-central Amazonia, Amazonas State, Brazil. Bold=type locality.

**Species**	**Latitude**	**Longitude**	**Municipality**	**Type of record**	**Source**
Mico acariensis	-5,118889	-60,020556	Novo Aripuanã	inference	Roosmalen et al., 2000
Mico acariensis	-6,900000	-59,050000	Apuí	observation	Noronha et al., 2007
Mico acariensis	-7,283333	-58,833333	Apuí	observation	Noronha et al., 2007
Mico acariensis	-6,798541	-59,055512	Apuí	observation	this paper
Mico acariensis	-6,814131	-59,077342	Apuí	observation	this paper
Mico acariensis	-7,085014	-59,629900	Apuí	observation	this paper
Mico chrysoleucos	-4,400000	-59,583333	Borba	observation	Wagner, 1842
Mico chrysoleucos	-3,300000	-58,250000	Itacoatiara	observation	Hershkovitz, 1977
Mico chrysoleucos	-3,366666	-58,750000	Itacoatiara	observation	Hershkovitz, 1977
Mico chrysoleucos	-3,383000	-58,260000	Itacoatiara	observation	Hershkovitz, 1977
Mico chrysoleucos	-4,360000	-59,716000	Borba	observation	Hershkovitz, 1977
Mico chrysoleucos	-7,260000	-60,380000	Apuí	observation	Hershkovitz, 1977
Mico chrysoleucos	-4,016414	-59,100000	Borba	observation	Silva-Júnior and Noronha, 2000
Mico chrysoleucos	-3,350556	-58,296678	Itacoatiara	observation	Silva et al., 2018
Mico chrysoleucos	-3,390856	-57,987231	Maués	observation	Silva et al., 2018
Mico chrysoleucos	-3,790836	-59,041389	Nova Olinda do Norte	observation	Silva et al., 2018
Mico chrysoleucos	-4,368075	-59,710858	Borba	observation	Silva et al., 2018
Mico chrysoleucos	-4,387525	-59,593636	Borba	observation	Silva et al., 2018
Mico chrysoleucos	-7,104853	-60,001692	Apuí	observation	Silva et al., 2018
Mico chrysoleucos	-7,104853	-60,645672	Novo Aripuanã	observation	Silva et al., 2018
Mico chrysoleucos	-7,233897	-60,007789	Apuí	observation	Silva et al., 2018

**Species**	**Latitude**	**Longitude**	**Municipality**	**Type of record**	**Source**
Mico chrysoleucos	-7,275289	-60,453353	Apuí	observation	Silva et al., 2018
Mico chrysoleucos	-7,533814	-60,667919	Apuí	observation	Silva et al., 2018
Mico chrysoleucos	-7,708381	-60,582564	Apuí	observation	Silva et al., 2018
Mico chrysoleucos	-8,358336	-59,864186	Apuí	observation	Silva et al., 2018
Mico chrysoleucos	-8,359453	-59,853911	Apuí	observation	Silva et al., 2018
Mico chrysoleucos	-2,948525	-58,060408	Urucurituba	observation	this paper
Mico chrysoleucos	-2,952100	-58,068181	Urucurituba	observation	this paper
Mico chrysoleucos	-3,337431	-57,886325	Maués	observation	this paper
Mico chrysoleucos	-5,225028	-60,230722	Novo Aripuanã	observation	this paper
Mico chrysoleucos	-7,204571	-60,018139	Apuí	vocalization	this paper
Mico chrysoleucos	-7,256651	-60,061049	Apuí	observation	this paper
Mico chrysoleucos	-7,466389	-60,521506	Apuí	observation	this paper
Mico chrysoleucos	-7,523015	-60,652183	Apuí	observation	this paper
Mico chrysoleucos	-7,697614	-60,619703	Apuí	observation	this paper
Mico chrysoleucos	-7,771944	-60,381639	Apuí	vocalization	this paper
Mico chrysoleucos	-8,236039	-60,034817	Apuí	observation	this paper
Mico chrysoleucos	-7,106933	-59,691281	Apuí	vocalization	this paper
Mico melanurus	-8,350000	-58,616667	Apuí	observation	Noronha et al., 2008
Mico melanurus	-8,566667	-59,133333	Apuí	observation	Noronha et al., 2008
